# Generation of New Isogenic Models of Huntington’s Disease Using CRISPR-Cas9 Technology

**DOI:** 10.3390/ijms21051854

**Published:** 2020-03-08

**Authors:** Magdalena Dabrowska, Agata Ciolak, Emilia Kozlowska, Agnieszka Fiszer, Marta Olejniczak

**Affiliations:** 1 Department of Genome Engineering, Institute of Bioorganic Chemistry, Polish Academy of Sciences, Noskowskiego 12/14, 61-704 Poznan, Poland; mdabrowska@ibch.poznan.pl; 2 Department of Medical Biotechnology, Institute of Bioorganic Chemistry, Polish Academy of Sciences, Noskowskiego 12/14, 61-704 Poznan, Poland; aluzna@ibch.poznan.pl (A.C.); emiliak@ibch.poznan.pl (E.K.); agnieszka.fiszer@ibch.poznan.pl (A.F.)

**Keywords:** genome editing, iPSCs, aberrant splicing, CAG repeats, Huntington’s disease, CRISPR

## Abstract

Huntington’s disease (HD) is a fatal neurodegenerative disorder caused by the expansion of CAG repeats in exon 1 of the huntingtin gene (*HTT*). Despite its monogenic nature, HD pathogenesis is still not fully understood, and no effective therapy is available to patients. The development of new techniques such as genome engineering has generated new opportunities in the field of disease modeling and enabled the generation of isogenic models with the same genetic background. These models are very valuable for studying the pathogenesis of a disease and for drug screening. Here, we report the generation of a series of homozygous HEK 293T cell lines with different numbers of CAG repeats at the *HTT* locus and demonstrate their usefulness for testing therapeutic reagents. In addition, using the CRISPR-Cas9 system, we corrected the mutation in HD human induced pluripotent stem cells and generated a knock-out of the *HTT* gene, thus providing a comprehensive set of isogenic cell lines for HD investigation.

## 1. Introduction

Huntington’s disease (HD) is an incurable and progressive neurodegenerative disease caused by the expansion of CAG repeats in exon 1 of the huntingtin gene (*HTT*) [1]. Depending on the number of CAG repeats, *HTT* alleles are classified as normal (<27), intermediate (28–35), reduced penetrance (36–39) and fully penetrant mutant alleles (>40) [2]. The most severe form of HD, known as juvenile HD, is associated with a >60 CAG expansion and onset before 20 years of age. Because HD is inherited in an autosomal dominant manner, patients harbor a single copy of mutant *HTT* encoding a huntingtin protein containing a polyglutamine (polyQ) domain [3]. The pathogenesis of HD is not fully understood; however, toxic gain-of-function resulting from mutant huntingtin is considered the most prominent cause [4,5,6]. During the disease course, loss of neurons in the striatum and cortex is observed, accompanied by reactive gliosis and astrocytosis, leading to progressive movement abnormalities and dementia. Different cellular models are being applied to study the pathogenesis of the disease and to test therapeutic approaches for HD. These models include HEK 293T cells with exogenous expression of mutant *HTT* or a fragment thereof, patient-derived fibroblasts, human and rodent induced pluripotent stem cells (iPSCs), neural stem cells (NSCs) and postmitotic neurons [7]. Healthy cells of different origins are generally used as controls in these experiments, increasing the risk of inappropriate interpretation of the observed phenotypes. The influence of the genetic background on the disease phenotype is increasingly being proven in HD, especially in light of the identification of genetic modifiers that affect the age of onset of HD in genome-wide association studies (GWAS) [8]. Subtle differences in DNA may influence phenomena such as somatic instability or disease onset.

Despite the monogenic nature of HD and the fact that its genetic basis was discovered more than two decades ago, we still do not understand all aspects of HD pathogenesis, such as the somatic instability of CAG repeats or the role of normal huntingtin in the adult brain and other tissues. This knowledge would be very important for the justification of allele-selective vs nonselective therapeutic approaches for this still incurable disease [9,10]. Rapidly developing genome editing tools such as CRISPR-Cas9 provide an opportunity to generate new isogenic models without background-related variability and to fill these knowledge gaps [11,12,13,14,15]. The CRISPR-Cas9 system uses the Cas9 nuclease and a small guide RNA (sgRNA) for the site-specific cleavage of a target sequence containing a protospacer-adjacent motif (PAM) [16]. Double-strand breaks (DSBs) are mainly repaired by nonhomologous end joining (NHEJ) or homology-directed repair (HDR) when the donor template is delivered. In this way, genes are being knocked-out or the mutation is repaired or introduced into specific *loci*.

Because human iPSCs (hiPSCs) can be differentiated into virtually every cell type, they serve as a valuable model for disease modeling and drug screening [7,17,18,19]. A few isogenic pairs of mutant/control hiPSC lines for HD have been established using traditional homologous recombination [20] and CRISPR-Cas9 technology [21,22]. The corrected cell lines were differentiated into neural progenitor cells (NPCs) and active neurons, and the reversal of HD-associated phenotypes was observed. However, one of these isogenic pairs contains 18/180 CAG repeats, which is an extremely long mutant variant [21], and the second pair contains 19/97 mixed CAG/CAA repeats [22], which does not reflect the typical sequence or length of the mutant tract in *HTT*. A panel of isogenic HD human embryonic stem cells (hESCs) with a more relevant repeat number (30, 45, 65 and 81 CAGs) was generated recently with the use of transcription activator-like effector nucleases (TALENs) [23]. These cell lines were differentiated into NPCs, neurons, hepatocytes and muscle cells. Transcriptomic and proteomic analyses identified cell-type and CAG-repeat length-dependent phenotypes.

Drug screening experiments are usually performed using exogenous reporter systems expressing *HTT* gene fragments in easy-to-transfect cells such as HEK 293 cells. This method is convenient and quantitative; however, it is simplified and “artificial” and does not include the potential influence of genomic context, promoter strength, or the full length gene. Endogenous models of HD such as patient-derived fibroblasts are sensitive to plasmid transfection and are therefore not useful for vector-based drug screening [24]. HEK 293 cells and hiPSCs can be expanded indefinitely and are popular cellular models for many studies. Using genome editing technology, the endogenous *HTT* locus can be modified by lengthening the CAG repeat tract in frame to produce mutant huntingtin [25].

Here, we used various approaches involving CRISPR-Cas9 technology to successfully generate new isogenic models of HD. A series of HEK 293T cell lines with different numbers of CAG repeats at the endogenous *HTT* locus was generated, and the usefulness of these lines in the testing of therapeutic reagents was demonstrated. In addition, isogenic controls for juvenile HD hiPSCs (19/109 CAG repeats) were generated. These cell lines exhibit a normal-length CAG tract (19/19 CAG) in *HTT*. A hiPSC line with a knock-out of the *HTT* gene was also obtained. These cell lines were characterized in detail and can be used as valuable models to study the pathogenesis and therapy of HD.

## 2. Results

### 2.1. Generation of HEK 293T-Based Models of HD

**Figure 1:**
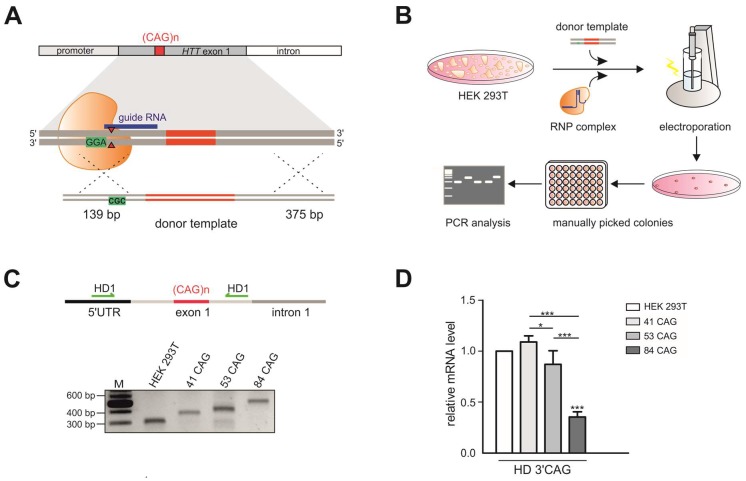

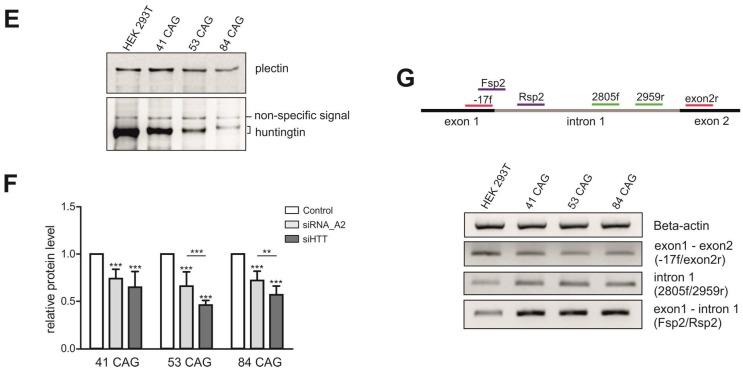
Generation of HEK 293T monoclonal cell lines with different numbers of CAG repeats at the *HTT locus*. (**A**) Successful strategy used to generate three monoclonal cell lines: 41 CAG, 53 CAG and 84 CAG. HTT_sg3 and SpCas9 were used to create DSB upstream from the CAG repeats. A linearized plasmid containing 41, 53 or 84 CAG repeats and silent mutation in a PAM sequence that doesn’t lead to an amino acid change (marked in green) served as a donor template for HDR. (**B**) Schematic representation of procedures used to generate HEK 293T cells with biallelic mutation at the *HTT locus*. (**C**) RT-PCR analysis of the edited *HTT* gene fragment containing 41, 53 or 84 CAG repeats. The PCR product comprises CAG repeat tract. Unmodified HEK 293T cells contain 16/17 CAG repeats at the *HTT locus*. (**D**) RT-qPCR analysis of the *HTT* mRNA level in 41 CAG, 53 CAG and 84 CAG cell lines. PCR primers were located downstream (3′ CAG) from the CAG repeats. All samples were normalized to β-actin, and the results are the mean (± SEM) relative to unmodified HEK 293T cells (*n* = 3; one-way ANOVA followed by Bonferroni’s post hoc test; * *p* = 0.01 to 0.05, ** *p* = 0.001 to 0.01, *** *p* < 0.001). (**E**) Western blot analysis of the huntingtin level in the edited cell lines and (**F**) cells transfected with the siRNA_A2 and siHTT. Plectin was used as a loading control. The results indicate the mean (± SEM) relative to cells treated with the control siRNA (BlockIT) (*n* = 3; one way ANOVA followed by Bonferroni’s post hoc test; *** *p* < 0.001). (**G**) Aberrant splicing of *HTT* mRNA results in appearance of abnormal transcript containing early intron 1. Analysis of the early intron 1 transcript by RT-PCR assays with three primer pairs spanning the exon 1–exon 2 junction (-17f/exon2r), the exon 1–intron (Fsp2/Rsp2) and intron 1 sequences (2805f/2959r). β-actin was used as a loading control.

To generate a model that is useful for studying the influence of repeat tract length on various aspects of the pathogenesis and therapy of HD, we generated HEK 293T cell lines with different numbers of CAG repeats at the *HTT* locus. To induce DSBs, we used previously validated sgRNAs [26] and different strategies: (i) Cas9 nickase (Cas9n) and an sgRNA pair (HTT_sg1 and HTT_sg4) flanking the repeat tract, (ii) wtCas9 or Cas9n and one sgRNA (HTT_sg4) targeting the sequence downstream of the CAG tract, (iii) wtCas9 and sgRNA (HTT_sg3) targeting the sequence located upstream of the repeat tract and (iv) a ribonucleoprotein (RNP) complex composed of the Cas9 protein and HTT_sg3 RNA. These strategies are summarized in Table S1. Only the last strategy resulted in frequent HDR and the generation of modified cell lines, which presented 41 CAG, 53 CAG or 84 CAG repeats (Figure 1A,B). Interestingly, biallelic modifications were more frequent than mono-allelic modifications. In heterozygous clones, the sequence of the mutant alleles was corrected without any scars (by HDR), whereas normal alleles contained indel mutations at a site of Cas9 cutting (resulting from NHEJ). We characterized the clonal cell lines by PCR amplification of the *HTT* gene region encompassing the CAG repeats (Figure 1C) and by analysis of huntingtin transcript and protein levels. Generally, the *HTT* transcript level measured by RT-qPCR with a primer pair located downstream of the CAG tract was correlated with the length of the CAG tract (Figure 1D). Proteins detected by western blotting migrated in polyacrylamide gels according to polyQ domain length, and the signal intensity was inversely correlated with the length of the polyQ tract, which is typically observed (Figure 1E). The sequences of the selected clones were confirmed by Sanger sequencing (Figure S1).

The generated HD models with increasing numbers of CAG tracts may serve as useful HD models for drug screening, especially using RNAi-based CAG-targeting strategies. Therefore, we transfected the edited HEK 293T cells with two previously validated siRNAs targeting either the exon 1 sequence under a non-allele-selective strategy (siHTT) [27] or the CAG tract under an allele-selective strategy (siRNA_A2) [28]. We analyzed the efficiency of silencing 48 h post-transfection by western blotting (Figure 1F, Figure S2). The level of the HTT protein was reduced by ~50% and ~30% in all models by siHTT and siRNA_A2, respectively (Figure 1F).

Pathogenic process in HD involves the production of smaller, N-terminal fragments of HTT [29] and some of this toxic products result from CAG repeat length–dependent aberrant splicing of exon 1 *HTT* [30,31]. To detect the early intron 1 transcripts, we used nonquantitative RT-PCR assays with three primer pairs spanning the exon 1–exon 2 junction, the exon 1–intron and intron 1 sequences (Figure 1G). The early intron 1 transcripts were elevated in HEK 293T cells which presented 41 CAG, 53 CAG or 84 CAG repeats compared to unmodified HEK 293T cells.

### 2.2. Correction of Mutation in HD Patient-Derived hiPSCs

To correct mutation in HD hiPSCs and generate isogenic control lines, we used CRISPR-Cas9 technology and a well-characterized patient-derived iPSC clonal line (ND42222, 19/109 CAG repeats in *HTT*). Because homologous recombination (HR) occurs with low efficiency, we used various genome-editing approaches and tools to induce DSBs and HDR. These strategies are summarized in Table S1. The first strategy involved a pair of sgRNAs (HTT_sg1 and HTT_sg4) and Cas9n expressed from a plasmid (described also in [32]). As a donor template, we used a 180 nt single-stranded oligodeoxynucleotide (ssODN) containing 10 CAG repeats. The electroporation efficiency was very low, and we observed cell death. This strategy resulted in a lack of correction by HDR, as we identified cell clones with CAG tract excision and perfect strand rejoining. The modification of the ssODN donor template by the extension of homologous arms and the introduction of a silent mutation in a PAM to avoid the recutting of the corrected alleles did not result in genetically corrected clones. In the next strategy, we used RNP complexes composed of the Cas9 protein and synthetic sgRNA targeting the *HTT* sequence upstream of the CAG tract (HTT_sg3). As a donor template, we used a shorter variant (180 nt) of ssODN. At 4 h before electroporation, cells were treated with nocodazole, which arrests S/M phases of the cell cycle and increases the frequency of HDR events. Among the 163 colonies screened, five were modified by HDR and contained 10/19 CAG, 19/19 CAG or 109/109 CAG repeats in *HTT*, but all of them except for the 19/19 CAG colony contained indel mutations at the cut site. HD hiPSCs were also electroporated with the Cas9 RNP and the HR donor plasmid carrying exon 1 of the *HTT* gene containing 19 CAG repeats (and silent mutation in a PAM). This strategy proved to be the most efficient; among the 131 colonies screened, eight were genetically corrected and contained 19/19 CAG repeats at the *HTT* locus. Three clonal cell lines were selected for more detailed analysis.

**Figure 2:**
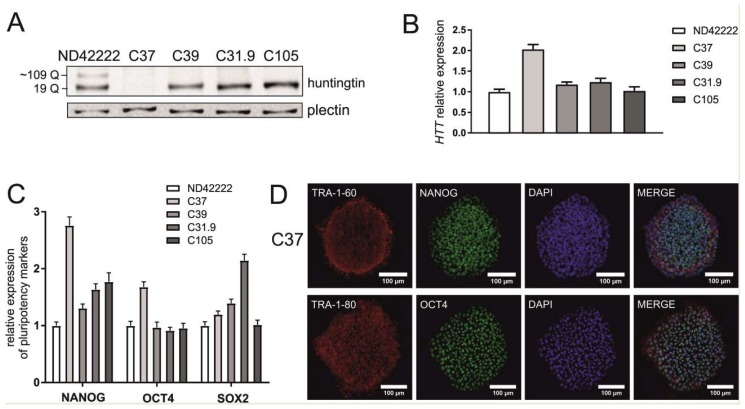
Characteristics of isogenic hiPSC clones. (**A**) Huntingtin protein level in HD hiPSC line with 19/109 Qs and its isogenic control cell lines was analyzed by western blotting. Plectin was used as a reference protein. (**B**) Analysis of the relative expression of the *HTT* gene by RT-qPCR in parental hiPSCs and generated cell lines. (**C**) The gene-edited hiPSCs maintain pluripotency as shown by expression of *NANOG*, *OCT4* and *SOX2* genes and (**D**) positive immunostaining for the pluripotency markers (presented for the C37 clonal cell line). The RT-qPCR results indicate the mean (± SEM) relative to expression level in ND42222, set at 1. ND42222 – parental HD hiPSCs; isogenic cell lines: C37 – HTT_KO hiPSCs, C39 – HD_19/19mut hiPSCs, C31.9 and C105 - HD_19/19 hiPSCs.

The newly generated isogenic control iPSCs were characterized by Sanger sequencing, *HTT* transcript and protein detection, the verification of pluripotency marker expression and karyotype analysis. These lines included iPSCs containing 19/19 CAG repeats (HD_19/19; clones C31.9 and C105), a similar cell line with a silent mutation present in one allele (HD_19/19mut; clone C39) and iPSCs with a double knock-out of the *HTT* gene (HD_KO; clone C37), which was achieved *via* the-out-of-frame excision of the CAG repeat tract with Cas9n, HTT_sg1 and HTT_sg4. Sequencing confirmed the purity of the clones, the repeat tract length and the presence of the silent mutation in C39 clone (Figure S3). This mutation results from homologous recombination with a donor plasmid carrying the silent mutation in a PAM. By western blotting, we confirmed either the presence of normal huntingtin in HD_19/19 (C31.9 and C105 clones) and HD_19/19mut (C39 clone) iPSCs or the absence of huntingtin in HD_KO iPSCs (C37 clone) (Figure 2A). Expression of *HTT* transcript was confirmed by RT-qPCR (Figure 2B). A lack of the most commonly occurring karyotypic abnormalities was observed in most of generated cell lines, as indicated by qPCR-based analysis (Figure S4). The exception was clone C31.9 with possible amplification of analyzed region at chromosome 4. *NANOG*, *OCT4* and *SOX2* markers showed similar expression level in generated lines, compared to that in parental hiPSCs, as analyzed by RT-qPCR (Figure 2C). Expression of pluripotency markers was also confirmed by positive staining for the nuclear markers OCT4 and NANOG and the surface proteins TRA 1–80 and TRA 1–60 (Figure 2D, Figure S5).

To study the potential off-target effects of the CRISPR-Cas9 system, we performed whole-exome sequencing (WES) of three isogenic control hiPSC clones (C37, C39 and C31.9) and a parental patient-derived iPSCs (ND42222) (Table S2). We did not observe mutations resulting from genome editing. A low number of single nucleotide variants (SNVs) were detected in corrected clones (Table S3) with 26 SNVs common to all three cell lines (Table S4).

## 3. Discussion

Here, we demonstrate the generation of new models of HD based on HEK 293T and hiPSCs. We employed a number of genome-editing approaches and tools, such as wtCas9 and Cas9n, different sgRNAs expressed from plasmids or delivered as RNP complexes, and different HR donor templates in the form of ssODNs or plasmid-based templates. A method involving the use of RNP complexes and an HR donor plasmid with a silent mutation in a PAM sequence was the most efficient in the case of both cell models. This strategy is safe and reduces the possibility of off-target mutations due to the short-term activity of the Cas9 protein and sgRNA delivered in an RNP complex. To induce DSBs, we used HTT_sg3 and the 5′AGG3′ PAM sequence located ~20 nt upstream of the CAG tract. In our previous study using the qEva-CRISPR assay, we demonstrated that HTT_sg3 did not induce nonspecific modifications in the tested off-target regions [32]. Other strategies were less efficient in the generation of successful edits; e.g., the use of double nickase strategy resulted in the preferential excision of CAG repeats, strand rejoining and *HTT* knock-out.

In our study, a series of isogenic HEK 293T cell clones containing expanded CAG repeats at the *HTT* locus was generated for the first time. The lengths of the repeat tracts represent frequent variants observed in HD patients (41 and 53 CAG repeats) and in a juvenile form of HD (84 CAG repeats). It is worth noting that each clone has a homozygous genotype in which the two alleles harbor a repeat tract of the same length. This characteristic makes these cell lines very useful considering the technical difficulties encountered in methods related to repeated sequences, as observed in PCR, sequencing and western blotting.

HEK 293 cells present some characteristics of neuronal lineage cells, such as the potential to propagate highly neurotropic viruses and inducible synaptogenesis [33]. It has been demonstrated that edited HEK 293 cells containing ~100 and 150 CAG repeats at the *HTT* locus undergo a wide spectrum of pathological changes characteristic of HD [25]. Despite the fact that HEK 293T cells differ significantly from neurons, which are the main site of HD pathogenesis, we observed the presence of the abnormal transcript resulting from the aberrant splicing of *HTT* mRNA. The same transcripts were detected in patient-derived fibroblasts, postmortem HD brains and mouse models expressing mutant *Htt* (mouse) or *HTT* (human) [31]. Nonetheless, HEK 293T cells, even with mutation in *HTT* gene, are not a good model to study some aspects of HD pathogenesis. The set of generated models will be valuable rather to study CAG repeat expansion/contraction mechanisms, aberrant splicing of *HTT* transcript, RAN translation, frameshifting or to test various huntingtin lowering therapeutic strategies. More appropriate models to study HD pathogenesis are neural cells derived from isogenic hiPSCs containing the same genetic background.

Using a similar strategy involving the CRISPR-Cas9 system delivered in an RNP complex and HR donor plasmid, we corrected HD iPSCs and generated healthy isogenic controls. In addition, by using an sgRNA pair and Cas9n, we excised the CAG repeat tract and generated a double knock-out of the *HTT* gene in parental HD iPSCs. HDR was inefficient under this approach. In a previous study involving the generation of isogenic HD control hiPSCs [21], the authors used Cas9n, a pair of sgRNAs and a piggyBac (PB) transposon selection cassette-based HR donor template. After the puromycin selection of edited clones and the excision of the selection cassette by transient transfection with a PB-expressing plasmid, 4.7% of clones were positively verified by immunoblotting. In our study, we used a much simpler approach involving the Cas9/sgRNA RNP complex without the need for additional selection steps, achieving a similar efficiency of editing (6%). We confirmed that the modified isogenic hiPSC clones retain pluripotency and a normal karyotype, and we demonstrated the expression of normal *HTT* in corrected control hiPSCs. Whole-exome sequencing demonstrated variability mainly due to clonal differences and the method imperfections, as we did not identify potential off-target sites in any of the lists of detected variants.

The parental HD hiPSC line ND42222 was recently characterized in detail using transcriptomics and proteomics approaches [34]. A number of deregulated genes were identified compared to healthy control hiPSCs. It would be interesting to validate these data by using isogenic controls to exclude the effects of the genetic background. Moreover, after the neural differentiation of hiPSCs, a set of isogenic cell lines, including a mutant line, a normal line and an *HTT* knock-out line, will be a valuable model for studying various aspects of HD.

## 4. Materials and Methods

### 4.1. HEK 293T Cell Culture And siRNA Transfection

HEK 293T cells containing 16/17 CAG repeats in the *HTT* gene (ATCC, Manassas, VA, USA) were grown in Dulbecco’s modified Eagle’s medium (Lonza; Basel, Switzerland) supplemented with 10% fetal bovine serum (FBS) (Sigma-Aldrich, St. Louis, MO, USA), antibiotics (Sigma-Aldrich) and l-glutamine (Sigma-Aldrich). All RNA oligonucleotides were synthesized at Future Synthesis (Poznan, Poland). Briefly, RNAs were combined in annealing buffer (Thermo Fisher Scientific, Waltham, MA, USA) to a 20-µM duplex concentration and incubated at 90 °C for 1 min, followed by additional incubation at room temperature for 45 min. The sequences of the siRNAs used in this study are presented in [27,28]. At 24 h prior to transfection 3 × 10^5^ cells were seeded on 6 cm plates. Cells transfections were performed using 100 nM siRNAs and Lipofectamine 2000 (Thermo Fisher Scientific) according to the manufacturer’s instructions. The transfection efficiency was monitored using 20 nM BlockIT fluorescent siRNA (Life Technologies, Carlsbad, CA, USA). Due to the rapid growth of the 41 CAG, 53 CAG and 84 CAG cell lines, the medium was changed after 4 h from transfection for the complete medium containing 4% FBS. The efficiency of silencing was analyzed 48 h post transfection by western blotting.

### 4.2. Donor Template

Single-stranded oligodeoxynucleotides (ssODNs) were synthesized (IDT, Skokie, IL, USA). The HR donor plasmid was prepared by cloning the PCR products with 41 and 85 CAG repeats (for HEK 293T cells) and 19 CAG repeats (for hiPSCs) and asymmetric homologous arms (139 bp and 375 bp) into the pGEM-T easy vector (Promega, Madison, WI, USA). During the transformation of GT116 *E. coli* cells, 85 CAG repeats were shortened to 53 and 84 CAG repeats. Finally, plasmids with 41, 53 and 84 CAG repeats (for HEK 293T) or 19 CAG repeats (for hiPSCs) were digested with the SacII enzyme (Thermo Fisher Scientific) and used as donor templates for further experiments. The PAM sequence in the donor template was mutated (5′AGG3′ to 5′ACG3′) using the QuikChange II XL Site-Directed Mutagenesis Kit (Agilent, Santa Clara, CA, USA) and mutHDg3F/mutHDg3R primers, to avoid nonspecific cutting of the plasmid by CRISPR-Cas9. To increase the frequency of HDR, cells were synchronized and arrested in G2/M phase by using 40 nM nocodazole (Sigma-Aldrich) at 4 h before electroporation.

### 4.3. HTT Gene Editing with the Plasmid–Based CRISPR-Cas9 System

The guide RNAs specific for the *HTT* gene (HTT_sg1, HTT_sg3 and HTT_sg4) were previously described and validated [26,32,35]. The top and bottom strands of the 20-nt guide RNAs (Table S5) were synthesized (IBB, Warsaw, Poland), annealed and ligated into the pair of FastDigest Bpil (Thermo Fisher Scientific) cut plasmids: pSpCas9(BB)-2A-GFP (PX458) and its nickase version (D10A nickase mutant; pSpCas9n(BB)-2A-GFP (PX461)) (Addgene, Cambridge, MA, USA) from *S. pyogenes* [36]. The ligated products were transformed into chemically competent *E. coli* GT116 cells (InvivoGen, San Diego, CA, USA), and the cells were plated onto ampicillin selection plates (100 μg/mL ampicillin) and incubated at 37°C overnight. Plasmid DNA was isolated using the Gene JET Plasmid Miniprep kit (Thermo Fisher Scientific) and verified with Sanger sequencing. Electroporation was used to deliver Cas9 protein, HTT_sgRNA and a donor template for HDR.

### 4.4. Editing of HEK 293T Cells with an RNP Complex

Cells were electroporated with an RNP complex composed of SpCas9, crRNA (HTT_sg3) and fluorescent tracer RNA, ATTO550, which is a novel fluorescent label related to the well-known dyes rhodamine 6G and rhodamine B (IDT), with 600 ng of a linearized HR donor plasmid. Before electroporation, CRISPR RNA (crRNA) and trans-activating small RNA (tracrRNA) oligos were mixed at an equimolar ratio in nuclease-free duplex buffer (IDT) to achieve a final concentration of the gRNA complex of 60 µM. The crRNA and tracrRNA duplex was heated at 95 °C for 5 min following 10 min of incubation at room temperature. The RNP complex was produced by mixing 5 μg (~30 pmol) of the recombinant NLS-SpCas9-NLS nuclease (VBCF Protein Technologies facility http://www.vbcf.ac.at) and 60 pmol of sgRNA, followed by incubation at room temperature for 10-20 min. HEK 293T cells were electroporated with the NeonTM Transfection System (Invitrogen, Carlsbad, CA, USA). Briefly, 2 × 10^5^ cells were harvested, resuspended in buffer R and electroporated with the RNP complex and 600 ng of the donor template in 10 μL tips using the following parameters: 1.150 V, 20 ms, 2 pulses. After electroporation, the cells were seeded at a low density (1–2 × 10^3^ cells/10 cm plate), and after 2 h, attached cells were identified based on the presence of a red signal (from fluorescent tracrRNA) during microscopic observation under a UV lamp. Monoclonal culturing was carried out for approximately 1.5 weeks, after which colonies were transferred to 48-well plates. The monoclonal cultures were analyzed after reaching approximately 60−80% confluency.

### 4.5. Generation of Human iPS Cells with Modifications in the HTT Gene

Parental HD iPSCs (ND42222) obtained from the NINDS Human Genetic Resource Center (Coriell Institute, Camden, NJ, USA) were characterized by NINDS via the analysis of pluripotency marker expression, colony formation, and karyotyping. Cells were grown in Essential 8 medium (Gibco, Thermo Fisher Scientific, Waltham, MA, USA) on Geltrex-coated dishes (Gibco). At 24 h before electroporation, after reaching 70% confluence, the medium was replaced with Essential 8 medium supplemented with the ROCK pathway inhibitor Y-27632 (STEMCELL Technologies, Vancouver, Canada) (final concentration 10 µM) to increase the survival of cells after electroporation. At 4 h before electroporation, the medium was supplemented with nocodazole (40 nM) [37,38]. Then, the cells were dissociated to single cells by incubation with 0.5 mM PBS-EDTA solution for 10 min. Next, 2 × 10^5^ cells were harvested, resuspended in buffer R and electroporated with the RNP complex and 600 ng of the donor template. Electroporation was conducted in 10 μL tips using the following parameters: 1100 V, 20 ms, and 2 pulses. After electroporation, the cells were seeded on Geltrex-coated dishes at low densities (2–15 × 10^3^ cells/10 cm plate). Four hours later, after the attachment of the cells, the medium was replaced with StemFlex Medium (Gibco) containing Y-27632. Then, cells exhibiting red signals were identified. The medium was replaced every 3–4 days until newly formed colonies exhibited sufficient growth for transfer. Monoclonal cultures derived from single iPSCs were manually picked and transferred to Geltrex-coated 48-well plates. Monoclonal cultures were grown in StemFlex Medium until they reached 80% confluence and then analyzed.

### 4.6. DNA Extraction and PCR

Genomic DNA from the HEK 293T and iPSC monoclones was extracted using QuickExtract™ DNA Extraction Solution (Lucigen, Middleton, WI, USA) according to the manufacturer’s instructions. Genomic DNA was amplified using GoTaq^®^ G2 DNA Polymerase (Promega) with primers HD1F and HD1R spanning the CAG repeats in exon 1 of the *HTT* gene. The PCR amplification program was as follows: initial denaturation at 95 °C for 3 min; 30 cycles at 95 °C for 30 s, 62 °C for 30 s, and 72 °C for 45 s; and a final elongation at 72 °C for 5 min. The same conditions were used for RT-PCR, with annealing temperature 59 °C for all primer pairs (−17f and Exon2r, 2805f and 2959r and Fsp2 and Rsp2). The PCR products were separated in 1.3% agarose gels and detected using UV transilluminator G:BOX (Syngene, Cambridge, UK). The PCR products of selected clones were purified using the GeneJET PCR Purification Kit (Thermo Fisher Scientific) and sequenced with the HD1F primer. The primer sequences are listed in the Table S6.

### 4.7. RNA Extraction and RT-qPCR

Total RNA was isolated from HEK 293T cells using the TRI Reagent (BioShop, Burlington, Canada) according to the manufacturer’s instructions. The RNA concentration was measured using a spectrophotometer (DeNovix, Wilmington, NC, USA). A total of 700 ng (HEK 293T) or 500 ng of RNA (hiPSCs) was reverse transcribed at 55 °C using Superscript III (Life Technologies) and random hexamer primers (Promega). The quality of the reverse transcription (RT) reaction was assessed through polymerase chain reaction (PCR) amplification of the β-actin gene. Complementary DNA (cDNA) was employed for quantitative polymerase chain reaction (qPCR) using SsoAdvanced™ Universal SYBR^®^ Green Supermix (Bio-Rad, Hercules, CA, USA) with denaturation at 95 °C for 30 s, followed by 40 cycles of denaturation at 95 °C for 15 s and annealing at 60 °C for 30 s. The melt curve protocol was subsequently performed with *HTT*-, pluripotency markers-, or *β-actin* or *GAPDH*-specific primers as follows: 5 s at 65 °C, followed by 5 s increments at 0.5 °C from 65 °C to 95 °C, in the CFX Connect™ Real-Time PCR Detection System (Bio-Rad). The primers used for RT-qPCR were designed to cover the *HTT* region downstream (HD 3′CAG) of the CAG repeat tract. The sequences of the primers are presented in Supplementary Table S6. Data preprocessing and normalization were performed using Bio-Rad CFX Manager software (Bio-Rad).

### 4.8. Immunocytochemistry

Generated iPSCs were plated on Matrigel (Corning, NY, USA)-coated cover slips and grown in Essential 8 medium. Then, the cells were fixed in 4% PFA, permeabilized with 0.5% Tween and blocked in 1% bovine serum albumin. Next, the cells were incubated overnight with the primary antibodies (listed in Table S7) at 4 °C. Thereafter, the cells were washed 3× with PBS for at least 5 min each time and incubated 1 h with fluorescent-dye conjugated secondary antibodies at room temperature and again washed 3× with PBS for at least 5 min. SlowFade Diamond Antifade Mountant with DAPI (Invitrogen) was used for nuclear staining. Images were captured on a Leica SP5 confocal microscope.

### 4.9. Western Blotting

Protein was isolated from the cells with the use of PB (60 mM Tris-base, 2% SDS, 10% sucrose, 2 mM PMSF). A total of 30 μg of protein was resolved on a Tris-acetate sodium dodecyl sulfate (SDS)-polyacrylamide gel (1.5 cm, 4% stacking gel/4.5 cm, 5% resolving gel, acrylamide:bis-acrylamide ratio of 49:1) in XT Tricine buffer (Bio-Rad) at 135 V in an ice-water bath. After electrophoresis, the proteins were wet-transferred overnight to a nitrocellulose blotting membrane (GE Healthcare Life Sciences, Chicago, Illinois, IL, USA). The primary antibodies, including anti-huntingtin, anti-plectin, and the anti-rabbit HRP conjugate secondary antibody were used in a PBS/0.1% Tween-20 buffer containing 5% nonfat milk. The immunoreaction was detected using the Westar Antares Chemiluminescent substrate (Cyanagen, Bologna, Italy). The protein bands were scanned directly from the membrane using a camera and quantified using a Gel-Pro Analyzer (Media Cybernetics, Rockville, MD, USA). A list of all antibodies used is provided in Table S7.

### 4.10. Karyotyping

Genomic DNA was isolated with a Genomic DNA Isolation Kit (Norgen Biotek, Schmon Parkway, Thorold, Canada). Then, a qPCR-based hPSC Genetic Analysis Kit (STEMCELL Technologies) was used to detect the major potential karyotypic abnormalities reported in human iPSCs according to the manufacturer’s instructions. Because the designed genome editing procedure was performed within chromosome 4, we used the Chr1q region as an internal control instead of the default region of Chr4p in calculations concerning potential abnormalities.

### 4.11. Whole-Exome Sequencing and Data Analysis

WES analysis was performed by CeGaT GmbH (Tubingen, Germany). 50 ng of high-molecular weight DNA per sample were used for preparing exome-enriched libraries with Twist Human Core Exome kit (Twist Bioscience, San Francisco, CA, USA). This kit is designed to target 33 Mb of highly conserved protein-coding regions. DNA fragmentation was performed using an enzymatic reaction. Subsequently, end-repair, dA-tailing, index adaptor ligation and purification was performed. These steps are followed by pre-capture PCR amplification and bead-based purification. The library was quantified using a Qubit dsDNA Broad Range Quantitation Assay and an average fragment length of 375 bp to 425 bp was ensured. Amplified, indexed libraries were pooled and hybridized to capture probes and bound to streptavidin binding beads. As a next step, post-capture PCR amplification was performed. After purification, the libraries were quantified using the Agilent BioAnalyzer High Sensitivity DNA Kit and a Thermo Fisher Scientific Qubit dsDNA High Sensitivity Quantitation Assay. Sequencing was performed on a NovaSeq 6000 (Illumina, San Diego, CA, USA) and 14.8 Gb (C31.9), 9.5 Gb (C39), 10.5 Gb (ND42222), and 12.2 Gb (C37) of data were produced. This resulted in an average coverage of 134, 91.7, 110.6, and 114.9, respectively.

Trimmed raw reads were aligned to the human reference genome (hg19-cegat) using the Burrows-Wheeler Aligner (BWA-mem version 0.7.17-cegat) [39]. ABRA (version 2.18) [40] was used for local realignment of reads in target regions to facilitate more accurate indel calling. In the reference hg19-cegat the pseudo-autosomal regions (PAR) on chromosome Y were masked (chrY:10001-2649520, chrY:59034050- 59363566). This procedure prevents reads that map to this region from being discarded due to mapping to two different chromosomes. Reads that could be aligned to more than one locus with the same mapping score were discarded. Duplicated reads, which most likely originated from the same PCR amplicon, were discarded as well. A proprietary software was used for variant detection (observed frequency of the alternative allele (OFA) > 0.85). Variants that occurred in the control sample ND42222 were excluded from the lists of the treated samples.

HTT_sg1, HTT_sg3 and HTT_sg4 were used to conduct an off-target site search using CCTop [41] allowing for up to four mismatches. Tables containing insertions and deletions as well as SNVs and the lists of potential off-target sites were imported into R version 3.6.1. Positions were then compared using the dplyr package. No position that was identified as a potential off-target site was present in any of the lists.

### 4.12. Mycoplasma Testing

Cultures were tested for mycoplasma contamination using a Veno GeM Classic Mycoplasma PCR detection Kit (Minerva Biolabs, Berlin, Germany) according to the manufacturer’s instructions.

### 4.13. Statistical Analysis

Statistical analysis was performed using GraphPad Prism v. 5.0 software (GraphPad, San Diego, CA, USA). Data were analyzed using one-way ANOVA followed by Bonferroni’s post hoc test (*p*-value: ns >0.05, * 0.01 to 0.05, ** 0.001 to 0.01, *** *p* < 0.001) with an arbitrary value of 1 assigned to the unmodified HEK 293T cells (Figure 1D) and to the cells treated with control (non-targeting) siRNA (Figure 1F).

## Supplementary Materials

Supplementary materials can be found at https://www.mdpi.com/1422-0067/21/5/1854/s1. Figure S1. Sanger sequencing analysis of the HTT locus in modified HEK 293T cell lines (41 CAG, 53 CAG and 84 CAG); Figure S2. Western blot analysis of HTT protein downregulation in edited HEK 293T cells treated with siRNA_A2 (A2) and siHTT. C – cells treated with control siRNA (without target). Plectin was used as a loading control; Figure S3. Sanger sequencing analysis of edited hiPSC lines; Figure S4. Results from qPCR-based analysis of potential karyotypic abnormalities in generated isogenic hiPSC lines. In case of C31.9 clone possible amplification of analyzed region at chromosome 4 is observed; Figure S5. The gene-edited hiPSCs maintain pluripotency as shown by positive immunostaining for the pluripotency markers; Table S1. Editing strategies used to generate isogenic models of HD in HEK 293T cells and hiPSCs; Table S2. Summary of capture statistics for whole-exome sequencing; Table S3. Sequence Variants in the gene-corrected hiPSC clones by whole-exome sequence analysis; Table S4. Sequence variants present in all edited clones C37, C39, C31.9 detected by whole-exome sequencing; Table S5. DNA and RNA oligonucleotides used for the generation of sgRNAs; Table S6. DNA oligonucleotides used as primers for PCR, RT-qPCR and directed mutagenesis; Table S7. Antibodies used for immunocytochemistry (ICC) and western blotting (WB).
