# Supplementary material for: Generation of New Isogenic Models of Huntington’s Disease Using CRISPR-Cas9 Technology
Source: Int J Mol Sci. 2020 Mar 8;21(5):1854. doi: 10.3390/ijms21051854 (PMC7084361; doi:10.3390/ijms21051854)
Supplement: Supplementary file 1 [file ijms-21-01854-s001.pdf]

Article

Generation of New Isogenic Models of Huntington's Disease Using CRISPR-Cas9 Technology

Magdalena Dabrowska [1], Agata Ciolak [2], Emilia Kozlowska [2], Agnieszka Fiszer [2] and Marta Olejniczak [1,*]

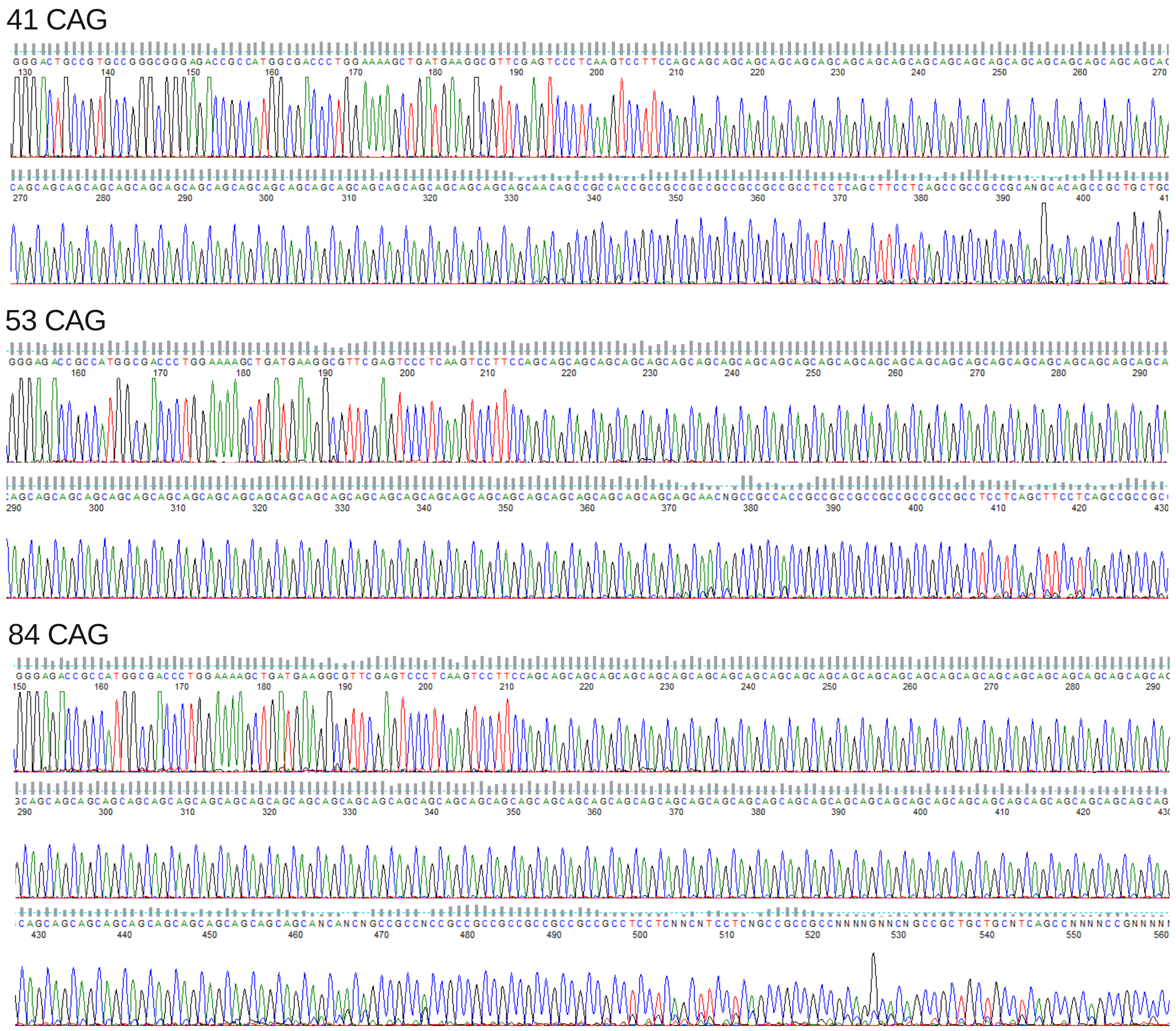

Supplemental Figure S1. Sanger sequencing analysis of the *HTT locus* in modified HEK 293T cell lines (41 CAG, 53 CAG and 84 CAG).

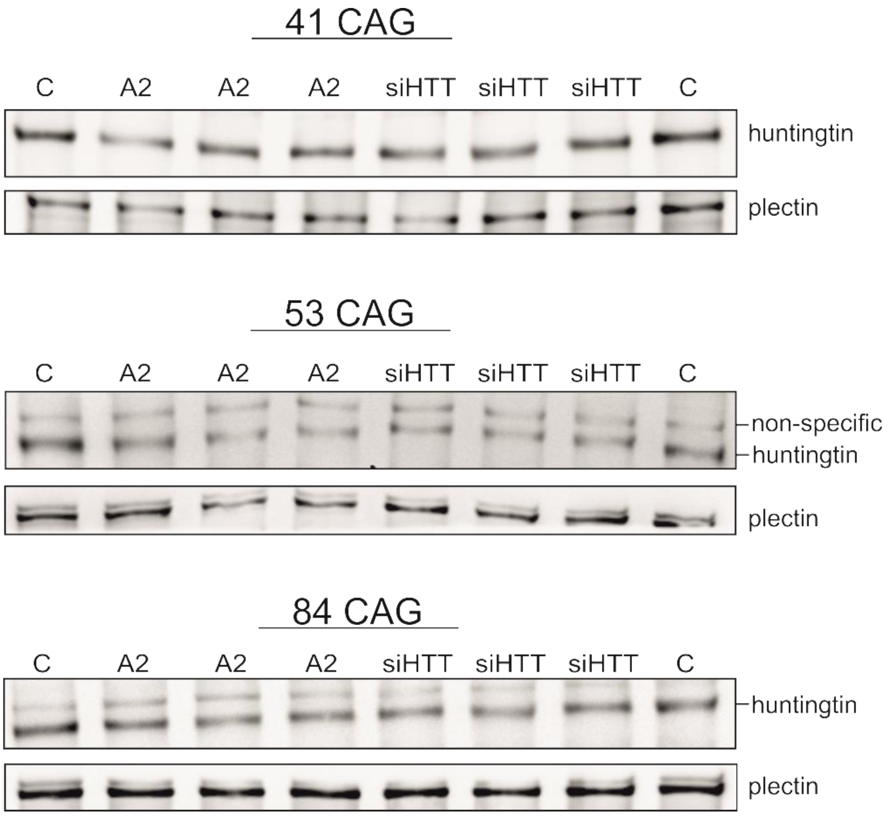

Supplemental Figure S2. Western blot analysis of HTT protein downregulation in edited HEK 293T cells treated with siRNA_A2 (A2) and siHTT. C – cells treated with control siRNA (without target). Plectin was used as a loading control.

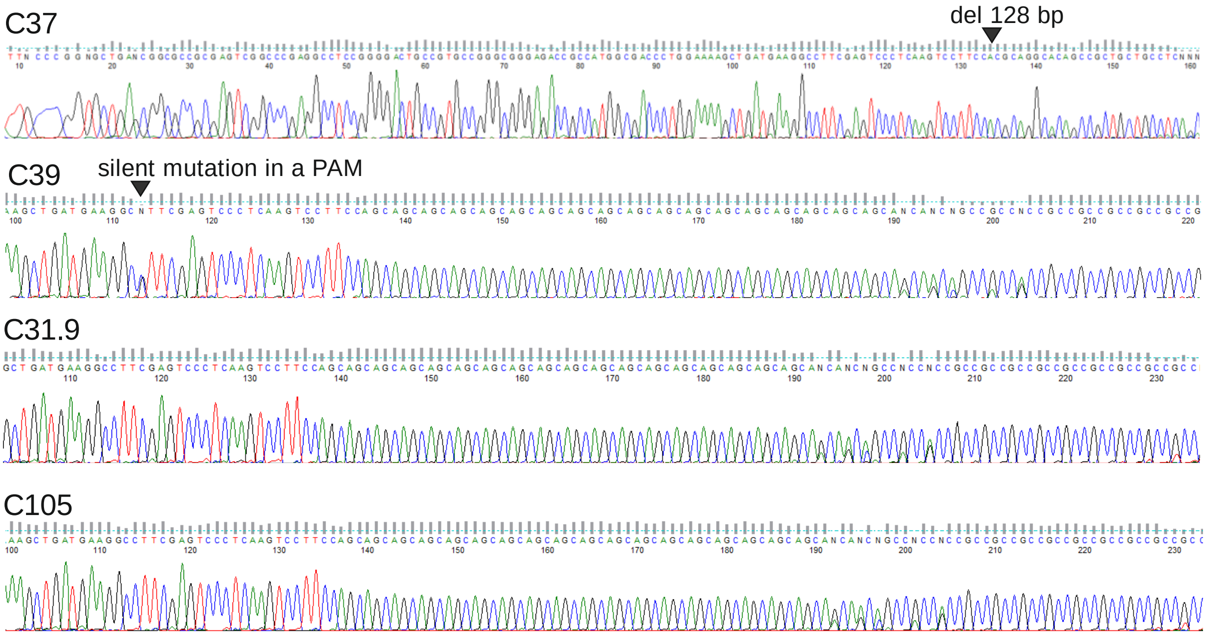

Supplemental Figure S3. Sanger sequencing analysis of edited hiPSC lines.

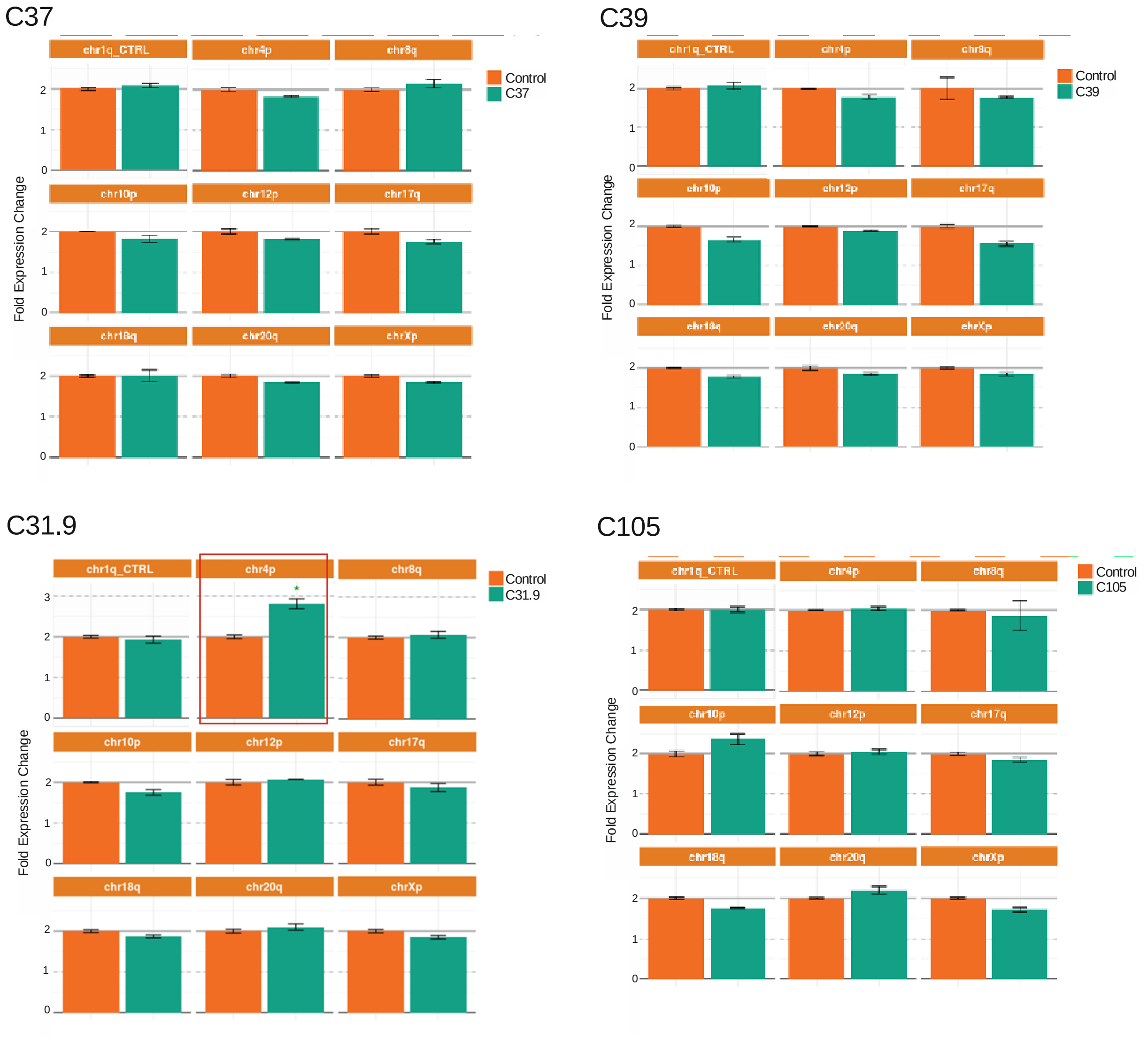

Supplemental Figure S4. Results from qPCR-based analysis of potential karyotypic abnormalities in generated isogenic hiPSC lines. In case of C31.9 clone possible amplification of analyzed region at chromosome 4 is observed.

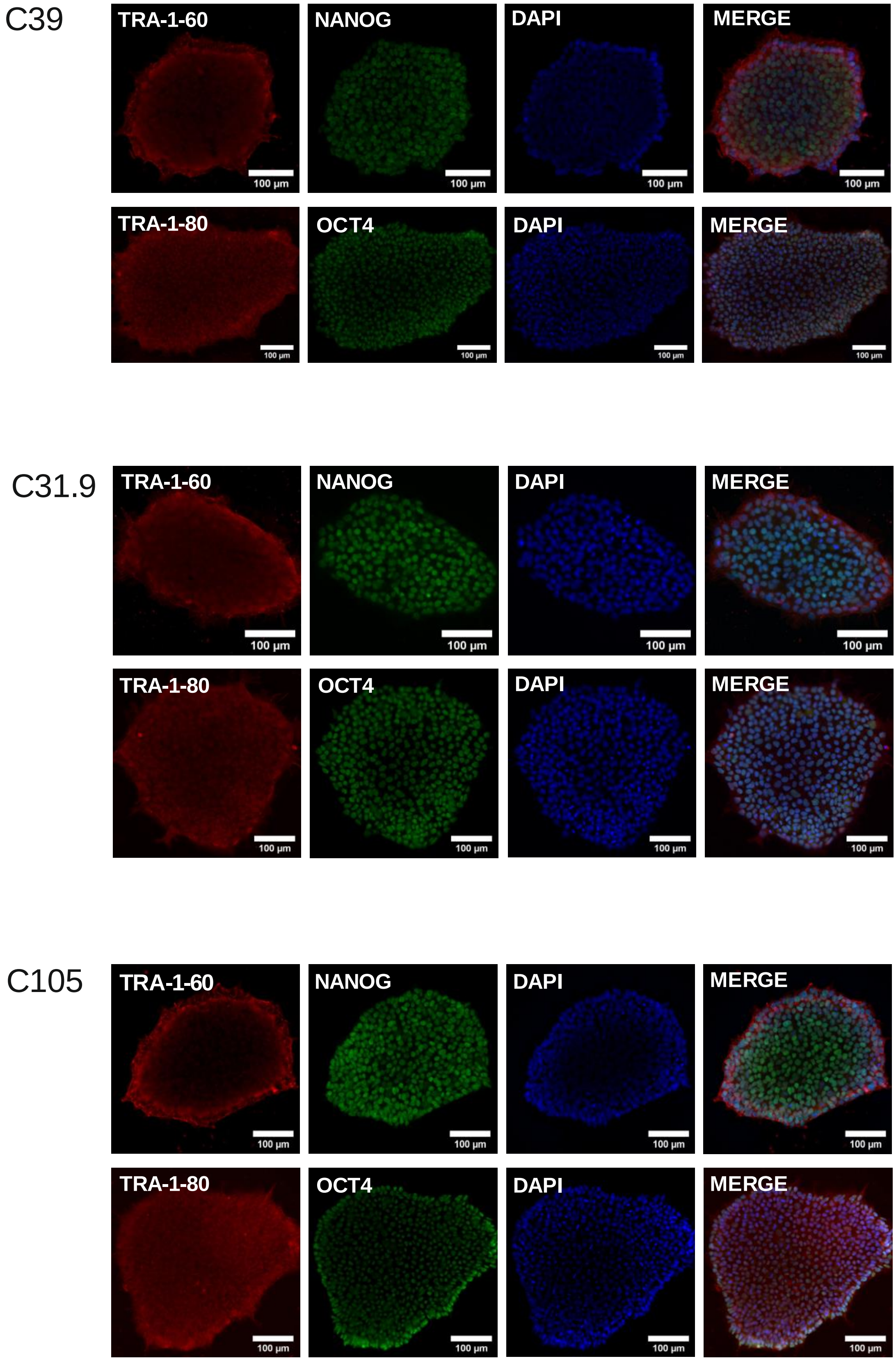

Supplemental Figure S5. The gene-edited hiPSCs maintain pluripotency as shown by positive immunostaining for the pluripotency markers.

Supplemental Table S1. Editing strategies used to generate isogenic models of HD in HEK 293T cells and hiPSCs.

**HEK 293T**					
	**Endonuclease**	**sgRNA**	**Donor template**	**HDR efficiency (%)**	**Results**
1	Cas9 nickase, plasmid	Pair: HTT_sg1 and HTT_sg4	ssODN (silent mutation in a PAM)	0	Indel mutations
2	Cas9 nickase, plasmid	HTT_sg4	ssODN (silent mutation in a PAM)	0	Indel mutations
3	Cas9 wt, plasmid	HTT_sg4	ssODN (silent mutation in a PAM)	0	Indel mutations
4	Cas9 wt, plasmid	HTT_sg4	Plasmid with exon 1 of the *HTT* gene	0	Indel mutations
5	Cas9 wt, plasmid	HTT_sg3	Plasmid with exon 1 of the HTT gene	7/109 (6.4%)	Homo- and heterozygous monoclones, all with indel mut in a cut site
6	Cas9 wt, protein	HTT_sg3, RNA	Plasmid with exon 1 of the *HTT* gene (silent mutation in a PAM)	41- 15/70 (21.42%)<br>53- 14/83 (16.87%)<br>84- 13/71 (18.31%)	Homozygous monoclones with 41, 53 and 84 CAG; (silent mutation in a PAM) and heterozygous monoclones all with indel mut in a cut site
**hiPSCs**					
1	Cas9 nickase, plasmid	Pair: HTT_sg1 and HTT_sg4	ssODN; 10 CAG, 180 nt	0	Low electroporation efficiency, apoptosis, CAG excision, strand rejoining
2	Cas9 nickase, plasmid	Pair: HTT_sg1 and HTT_sg4	ssODN (silent mutation in a PAM), longer arms, 300 nt	0	Low electroporation efficiency, apoptosis, CAG

					excision, strand rejoining
3	Cas9 wt, protein	HTT_sg3, RNA	ssODN<br>10 CAG, 18 0nt	0.61% (10mut/19CAG); 1.23% (19/19CAG); 1.23% (109/109 CAG)	163 monoclones in total
4	Cas9 wt, protein	HTT_sg3, RNA	Plasmid with exon 1 of the *HTT* gene (19 CAG; silent mutation in a PAM)	6% (19/19CAG)	131 monoclones in total

Supplemental Table S2. Summary of capture statistics for whole-exome sequencing.

**Sample ID**	**Mean Read Length**	**Total Reads (in million)**	**After Removing Identical Reads (in million)**	**Unique (%)**	**Mapped Reads (in million)**	**Mapping (%)**
ND42222	99	105,3	90,674	86,11	89,401	98,6
C37	99	123,391	104,87	84,99	102,94	98,16
C39	99	95,493	83,098	87,02	81,936	98,6
C31.9	99	149,826	122,902	82,03	120,54	98,08

Supplemental Table S3. Sequence Variants in the gene-corrected hiPSC clones by whole-exome sequence analysis

	**C37**	**C39**	**C31.9**
Total*	86	54	88
3_prime_UTR	0	0	0
3_prime_UTR_intronic	0	0	0
5_prime_UTR	3	1	1
5_prime_UTR_intronic	0	0	0
downstream_gene	0	0	0
essential_splice_site	0	0	0
initiator_codon	0	0	0
intronic	22	22	24
kozak_sequence	0	0	0
missense	31	15	29

non_coding_exonic	0	0	1
non_coding_intronic	0	0	1
splice_region	6	5	5
stop_gained	0	0	0
stop_lost	0	0	0
stop_retained	0	0	0
synonymous	24	11	27
upstream_gene	0	0	0

*OFA>0.85

Supplemental Table S4. Sequence variants present in all edited clones C37, C39, C31.9 detected by whole-exome sequencing.

Chr	Position	Gene	Ref	Alt	Consequence
chr1	220789362	MARK1	T	A	intronic
chr2	131220864	POTEI	T	A	missense
chr2	214012405	IKZF2	A	C	intronic
chr3	195506446	MUC4	G	T	missense
chr3	195510582	MUC4	A	C	synonymous
chr5	80756855	SSBP2	A	T	intronic
chr5	87502325	TMEM161B	G	A	intronic
chr6	18134021	TPMT	C	A	intronic
chr9	34725368	FAM205A	A	G	synonymous
chr10	29580942	LYZL1	C	T	splice_region
chr10	29580944	LYZL1	C	A	intronic
chr10	29580961	LYZL1	C	T	intronic
chr10	46321555	AGAP4	G	A	synonymous
chr12	10571716	KLRC3	A	T	intronic
chr12	10571716	AC068775.1	A	T	intronic
chr12	27826780	PPFIBP1	T	G	intronic
chr15	30906259	GOLGA8H	G	T	splice_region
chr15	32743481	GOLGA8O	C	T	intronic
chr15	69715488	AC027237.1	C	T	intronic
chr15	69715488	KIF23	C	T	intronic
chr16	71805160	AP1G1	G	A	chr16
chr19	7051376	MBD3L2	G	A	missense
chr19	43860251	CD177	G	A	chr19
chr19	43860255	CD177	T	G	missense

chr22	21797094	HIC2	C	G	5_prime_UTR
chr22	24300660	GSTT2B	G	C	intronic

Supplemental Table S5. DNA and RNA oligonucleotides used for the generation of sgRNAs

**Oligonucleotide**	**Sequence (5′-3′)**	**Description**
HTT_sg1s	**CACC**GCTGCTGCTGCTGCTGCTGGA	oligo for Cas9_HTT.sg1 plasmid construction
HTT_sg1a	**AAAC**TCCAGCAGCAGCAGCAGCAGC	oligo for Cas9_HTT.sg1 plasmid construction
HTT_sg3s	**CACC**GGAAGGACTTGAGGGACTCGA	oligo for Cas9_HTT.sg3 plasmid construction
HTT_sg3a	**AAAC**TCGAGTCCCTCAAGTCCTTCC	oligo for Cas9_HTT.sg3 plasmid construction
HTT_sg4s	**CACC**GGCTTCCTCAGCCGCCGCCGC	oligo for Cas9_HTT.sg4 plasmid construction
HTT_sg4a	**AAAC**GCGGCGGCGGCTGAGGAAGCC	oligo for Cas9_HTT.sg4 plasmid construction
HTT_sg3 RNA	IDT	tracrRNA, RNP strategy
HTT_sg3 crRNA	GAAGGACUUGAGGGACUCGA	crRNA, RNP strategy
ssODN	TGCCGTGCCGGGCGGGAGACCGCCATGGCGACCCT GGAAAAGCTGATGAAGGCCTTCGAGTCCCTCAAGT CCTTCCAGCAGCAGCAGCAGCAGCAGCAGCAGCAG CAACAGCCGCCACCGCCGCCGCCGCCGCCGCCGCC TCCTCAGCTTCCTCAGCCGCCGCCGCAGGCACAGCC GCTG	donor template for HDR

Supplemental Table S6. DNA oligonucleotides used as primers for PCR, RT-qPCR and directed mutagenesis.

Name	Forward (5′-3′)	Reverse (5′-3′)	Method
HD1	CCGCTCAGGTTCTGCTTTTA	GGCTGAGGCAGCAGCGGCTG	PCR, seq
−17f and Exon2r*	GAGCCGCTGCACCGAC	CTGACAGACTGTGCCACTATG TTT	PCR
2805f and 2959r*	GATTTTGGCAGTTCTGTTCAC G	ATAAACTGAGGCCCATGCAT G	PCR
Fsp2 and Rsp2	CTGCACCGACCGTGAGTT	CAAGGGAAGACCCAAGTGAG	PCR
HD 3′CAG	CGACAGCGAGTCAGTGATTG	ACCACTCTGGCTTCACAAGG	RT-qPCR
SOX2	CAAAAATGGCCATGCAGGTT	AGTTGGGATCGAACAAAAGC TATT	RT-qPCR
NANOG	TTTGGAAGCTGCTGGGGAAG	GATGGGAGGAGGGGAGAGGA	RT-qPCR
OCT3/4	AGTTTGTGCCAGGGTTTTTG	ACTTCACCTTCCCTCCAACC	RT-qPCR
Beta actin	TGAGAGGGAAATCGTGCGTG	TGCTTGCTGATCCACATCTGC	RT-qPCR
GAPDH	GAAGGTGAAGGTCGGAGTC	GAAGATGGTGATGGGATTTC	RT-qPCR
mutHDg3	GGAAAAGCTGATGAAGGCGT TCGAGTCCCTCAAGTC	GGACTTGAGGGACTCGAACG CCTTCATCAGCTTTTC	Directed mutagenesi s

*Sequences of primers are from Sathasivam K. et al., (2013) Proc Natl Acad Sci U S A, 110, 2366–2370

Supplemental Table S7. Antibodies used for immunocytochemistry (ICC) and western blotting (WB)

ICC	Antibody	Dilution	Company Cat # and RRID
Primary antibody (pluripotency markers)	Rabbit anti-OCT4	1:200	ThermoFisher Cat# PA5-27438 RRID: AB_2544914
	Rabbit anti-NANOG	1:200	Cell Signaling Cat#4903 RRID: AB_10559205
	Mouse anti-TRA-1-60	1:100	Millipore Cat# MAB4360 RRID: AB_2119183
	Mouse anti-TRA-1-80	1:100	ThermoFisher Cat#MA1-024 RRID: AB_2536706
Secondary antibody	Donkey Anti-Rabbit Alexa Fluor 488	1:1000	Jackson ImmunoResearch, West Grove, PA, USA Cat#711-546-152

			RRID: AB_2340619
Secondary antibody	Donkey Anti-Mouse Alexa Fluor 594	1:1000	Jackson ImmunoResearch Cat#715-586-151 RRID: AB_2340858
**WB**	**Antibody**	**Dilution**	**Company Cat # and RRID**
Primary antibody	Rabbit anti-huntingtin [EPR5526]	1:1000	Abcam, Cambridge, UK Cat#ab109115
Primary antibody	Rabbit anti-plectin	1:1000	Cell Signaling, Leiden, NED Cat#12254 RRID:AB_2797858
Secondary antibody	Anti-rabbit HRP-conjugate	1:2000	Jackson ImmunoResearch Cat# 711-035-152 RRID: AB_10015282
